# Molecular heterogeneity of anti-PD-1/PD-L1 immunotherapy efficacy is correlated with tumor immune microenvironment in East Asian patients with non-small cell lung cancer

**DOI:** 10.20892/j.issn.2095-3941.2020.0121

**Published:** 2020-08-15

**Authors:** Runsen Jin, Chengming Liu, Sufei Zheng, Xinfeng Wang, Xiaoli Feng, Hecheng Li, Nan Sun, Jie He

**Affiliations:** ^1^Department of Thoracic Surgery, National Cancer Center/National Clinical Research Center for Cancer/Cancer Hospital, Chinese Academy of Medical Sciences and Peking Union Medical College, Beijing 100021, China; ^2^Department of Thoracic Surgery, Ruijin Hospital, Shanghai Jiao Tong University School of Medicine, Shanghai 200025, China; ^3^Department of Pathology, National Cancer Center/National Clinical Research Center for Cancer/Cancer Hospital, Chinese Academy of Medical Sciences and Peking Union Medical College, Beijing 100021, China

**Keywords:** NSCLC, East Asian, oncogene mutations, PD-1/PD-L1 inhibitors, immune microenvironment

## Abstract

**Objective:** The aim of this study was to investigate how the tumor immune microenvironment differs regarding tumor genomics, as well as its impact on prognoses and responses to immunotherapy in East Asian patients with non-small cell lung cancer (NSCLC).

**Methods:** We performed an integrated analysis using publicly available data to identify associations between anti-programmed death 1 (PD-1)/ programmed death-ligand 1 (PD-L1) immunotherapy efficacy and classic driver oncogene mutations in East Asian NSCLC patients. Four pooled and clinical cohort analyses were used to correlate driver oncogene mutation status and tumor microenvironment based on PD-L1 and CD8^+^ tumor-infiltrating lymphocytes (TILs). Immune infiltrating patterns were also established for genomic NSCLC subgroups using the CIBERSORT algorithm.

**Results:** Based on East Asian NSCLC patients, TIDE analyses revealed that for anti-PD-1/PD-L1 immunotherapy, epidermal growth factor receptor (EGFR)-mutant and anaplastic lymphoma kinase (ALK)-rearranged tumors yielded inferior responses; however, although Kirsten rat sarcoma viral oncogene homolog (KRAS)-mutant tumors responded better, the difference was not statistically significant (EGFR: *P* = 0.037; ALK: *P* < 0.001; KRAS: *P* = 0.701). Pooled and clinical cohort analyses demonstrated tumor immune microenvironment heterogeneities correlated with oncogenic patterns. The results showed remarkably higher PD-L1- and TIL-positive KRAS-mutant tumors, suggesting KRAS mutations may drive an inflammatory phenotype with adaptive immune resistance. However, the EGFR-mutant or ALK-rearranged groups showed a remarkably higher proportion of PD-L1-/TIL-tumors, suggesting an uninflamed phenotype with immunological ignorance. Notably, similar to triple wild-type NSCLC tumors, EGFR L858R-mutant tumors positively correlated with an inflammatory phenotype, suggesting responsiveness to anti-PD-1/PD-L1 immunotherapy (*P* < 0.05). Furthermore, the CIBERSORT algorithm results revealed that EGFR-mutant and ALK-rearranged tumors were characterized by an enriched resting memory CD4^+^ T cell population (*P* < 0.001), as well as a lack of CD8^+^ T cells (*P* < 0.01), and activated memory CD4^+^ T cells (*P* = 0.001).

**Conclusions:** Our study highlighted the complex relationships between immune heterogeneity and immunotherapeutic responses in East Asian NSCLC patients regarding oncogenic dependence.

## Introduction

Non-small cell lung cancer (NSCLC) is one of the most common malignant tumors worldwide^1,2^. As the most common type of lung cancer, more than 40% of NSCLC patients are at the late stage at the time of diagnosis, and have missed their optimal opportunity for surgical tumor removal^3^. Recent advances in late stage lung cancer treatments include molecular targeted therapies and immunotherapy for managing NSCLC. The former depends on stratification and treatment based upon genetic mutations in oncogenic drivers, such as epidermal growth factor receptor (EGFR), anaplastic lymphoma kinase (ALK), and ROS proto-oncogene 1. Although targeted drugs such as tyrosine kinase inhibitors (TKI) achieve a marked effect, most patients develop resistance over time^4^. Remarkably, monoclonal antibodies such as pembrolizumab and nivolumab targeting the programmed death 1 (PD-1) receptor and its ligand programmed death-ligand 1 (PD-L1), facilitate a patient’s own T cells to kill tumors, resulting in remarkable antitumor activity in NSCLC patients^5,6^. Recent clinical trials^7–11^ and real-world data^12–14^ have shown that anti-PD-1/PD-L1 immunotherapy results in robust disease control, long-term survival, and improved quality of life in advanced NSCLC patients. Unfortunately, immunotherapy can only benefit a subgroup of patients, and demonstrates response rates of only 17%–21%^15^.

Several studies in Western countries have reported that NSCLC with EGFR-mutated or ALK-rearranged phenotypes have disappointing clinical outcomes, with lower objective response rates (ORRs) and shorter progression-free survival (PFS) to PD-1 /PD-L1 inhibitors^16^. Further subgroup analysis of clinical trials has indicated that patients with Kirsten rat sarcoma viral oncogene homolog (KRAS) mutations were more sensitive to PD-1 axis inhibitors, when compared to those with wild-type KRAS. Furthermore, using PD-1/PD-L1 inhibitors as second- or third-line therapy improved the overall survival (OS), when compared to standard chemotherapy in KRAS-mutant NSCLC patients^5,8,9,17^. A previous study also reported that patient outcomes may be optimized using molecular biomarkers recommended by The National Comprehensive Cancer Network (NCCN)^18^. Based on these observations, we hypothesized that the efficacy of anti-PD-1/PD-L1 immunotherapy varies according to different molecular phenotypes of the tumor. However, it was previously reported that lung cancer patients of Asian and Western countries differed in both histological types and genetic mutations^19^. In East Asia, at least 30% of the NSCLC patients have EGFR mutations as compared to < 10% in Western countries^20,21^. Moreover, the prevalence of East Asian patients with lung cancer that have KRAS mutations is about 5%–10%, compared to ≤ 35% of Caucasian patients^20,22,23^. Pan-cancer immunogenomic analyses have shown that tumor genotype largely determines their immunophenotype^24^. Thus, it is critical to investigate the correlation between anti-PD-1/PD-L1 immunotherapy efficacy and classic driver oncogene mutations in East Asian NSCLC patients.

Evidence has indicated that PD-L1 is generally upregulated in NSCLC patients, with PD-1 expressed on most tumor infiltrating lymphocytes (TILs), supporting the rationale for the development of monoclonal antibodies against PD-L1 or PD-1, which are currently under investigation. Preliminary results imply that PD-L1 positivity may correlate with a response to PD-1 pathway inhibitors^18^. Furthermore, several studies have suggested that oncogene activation could induce PD-L1 expression, representing innate immune resistance^25,26^. The expression of PD-L1 on tumor cells can be reflected either in an induced reaction to a T cell response, or a constitutive expression through oncogenic signaling^27^. In addition, Teng et al.^28^ reported a simplistic and pragmatic stratification of the tumor immune microenvironment (TIME) according to the presence of TILs and PD-L1 expressions, in which dual positive (PD-L1^+^/TIL^+^) tumors are most likely to benefit from anti-PD-1/PD-L1 antibodies. Given these observations, we hypothesized that based on PD-L1 expression and the presence of TIL, which impact anti-PD-1/PD-L1 immunotherapy efficacy, the TIME could vary based on the molecular phenotype of the tumor.

Although the understanding of the NSCLC immune landscape has largely improved, the relationship between tumor-infiltrating leukocytes and tumor genomics, and its impact on prognosis and response to immunotherapy, are still unclear. Notably, existing studies have been mainly based on data from Europe and the United States, with different lung cancer histological types and gene mutations in the East Asian population^19,29^. Thus, it is of great importance to deconvolute immune cell elements from bulk tissue gene expression profiles (GEPs) to determine the distinct immune cell composition in molecular subgroups of East Asian NSCLC patients.

In the present study, we characterized the association between classic driver oncogene mutations in East Asian NSCLC patients and the efficacy of anti-PD-1/PD-L1 immunotherapy, in addition to tumor immunity-associated features such as PD-L1 expression, the presence of CD8^+^ TIL, and the intratumoral immune cell composition. For the first time, our results revealed heterogenic responses to immunotherapy in East Asian NSCLC patients, which is correlated with the TIME according to not only PD-L1 expression and the presence of TIL, but also local repertoires of tumor-infiltrating leukocytes.

## Materials and methods

### Immunotherapeutic patients

Clinical information was downloaded for 207 NSCLC patients treated with anti-PD-1/PD-L1 monotherapy from cBioPortal (http://www.cbioportal.org/). The objective response to immune checkpoint blockades was assessed by Response Evaluation Criteria in Solid Tumors (RECIST), version 1.1, by a thoracic radiologist. Responders were patients with a confirmed complete or partial response, while those with stable disease, progressive disease, or not evaluable were considered to be non-responders. In addition to the response defined by RECIST, efficacy was also defined as durable clinical benefit (DCB, PFS > 6 months) or no durable benefit (NDB, PFS ≤ 6 months)^30^. Clinicopathological and molecular patient information and clinical outcomes are listed in **Supplementary Table S1**.

### Immunotherapeutic response prediction

We collected RNA-seq and corresponding clinical data of the GSE31210 dataset from the publicly available database, Gene Expression Omnibus (GEO, https://www.ncbi.nlm.nih.gov/geo/query/acc.cgi?acc=GSE31210). GSE31210 consists of 226 East Asian lung adenocarcinoma cases. Patient subtypes included 127 with EGFR mutations; 20 with KRAS mutations; 11 with ALK/EML4 fusions; and 68 without a common mutation. Transcriptome data were standardized across patients using the quantile-normalization method, and the expression value of each gene was normalized by subtracting the average among all samples, so a zero value indicated average expression.

Tumor Immune Dysfunction and Exclusion (TIDE, http://tide.dfci.harvard.edu/) was used to estimate TIDE prediction scores with normalized transcriptome data from each patient. Patients whose TIDE prediction scores were more than zero were considered responders; patients whose TIDE prediction scores were less than zero were considered non-responders^31^.

### Pooled analysis

Three pooled analyses investigated the possible correlations between PD-L1 expression and classic driver oncogene mutation status (EGFR mutation/KRAS mutation/ALK fusion) in resected East Asian NSCLC samples. The characteristics of patients from the included 23 studies, including region, stage, histology, PD-L1 antibody clone, and cut-off value, are presented in **Supplementary Table S2**. In addition, a pooled analysis was performed to investigate PD-L1 expression according to the mutant subtype in EGFR-mutant patients. The characteristics of patients from the five studies, including region, stage, histology, PD-L1 antibody clone, and cut-off value, are presented in **Supplementary Table S3**. Pooled analysis was performed independently by two authors (J.R.S. and L.C.M.) as previously described^32^.

### Clinical patients and immunohistochemical (IHC) analysis

The study collected 629 surgical specimens from NSCLC patients at the Cancer Hospital/Institute, Chinese Academy of Medical Sciences (CICAMS, Beijing, China) from January 2008 to December 2013. All samples were analyzed for classic driver oncogene mutation status, PD-L1 expression, and the presence of CD8^+^ TIL. Patients did not receive any preoperative treatments such as radiotherapy or chemotherapy, and no other tumors were diagnosed within 3 years before surgery. Clinical information of the cohort is listed in **Supplementary Tables S4 and S5**. The Ethics Committee of CICAMS approved this study. The approval number was CH-L-043. All enrolled patients signed the written informed consent form prior to the study, in accordance with the oversight of the local ethics committee.

The expression of PD-L1 and CD8A were detected by immunohistochemistry (IHC) using a PD-L1 SP263 assay (anti-human PD-L1 rabbit monoclonal antibody, #740-4907; Ventana Medical Systems, Tucson, AZ, USA) and a CD8A assay (anti-human CD8 rabbit monoclonal antibody, ZA-0508; Zsbio Tech, Beijing, China). All IHC slides were evaluated by two experienced pathologists blinded to the clinical parameters according to the evaluation criteria of prior methods^32^.

### Intratumoral immune cell composition analysis

CIBERSORT (https://cibersort.stanford.edu/) was used to calculate immune cell type fractions with the gene expression profile of each patient at 1,000 permutations, with the results further filtered using a value *P* < 0.05. Quantile normalization was used as recommended to remove confounding effects. Gene expression data of 226 East Asian lung adenocarcinoma cases, including 127 EGFR-mutant, 20 KRAS-mutant, 11 ALK/Echinoderm microtubule-associated protein-like 4 (EML4) fusion, and 68 without common mutation patients, were also collected from GSE31210.

### Statistical analysis

Prism software (version 5.0) (GraphPad, San Diego, CA, USA), Review Manager software (https://review-manager.software.informer.com/5.3/) (version 5.3), and R software (version 3.6.0) (The R Foundation for Statistical Computing, Vienna, Austria) were used to perform statistical analyses. According to the Cochrane handbook, we used Review Manager software to analyze statistical parameters in the pooled analyses^33^. Experimental data are reported as the mean ± SD. The chi-square test and the Mann-Whitney U test were used for statistical analyses between two different groups. The immune cell composition in each sample was compared among molecular subtypes of NSCLC using the Kruskal-Wallis test. Two-way analysis of variance was used for assessments among all groups. All represented *P* values were double-tailed, and a value of *P* < 0.05 was considered as statistical significance.

## Results

### Correlation between classic driver oncogene mutations and anti-PD-1/PD-L1 immunotherapy efficacy in NSCLC patients

**Figure 1:**
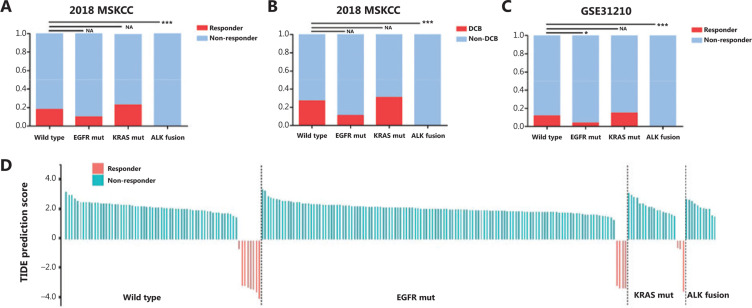
Correlation between the efficacy of anti-PD-1/PD-L1 immunotherapy and classic driver oncogene mutations in non-small cell lung cancer (NSCLC) patients. (A and B) Box plots evaluating objective response rate (A) and durable clinical benefit (progression-free survival > 6 months) (B) of NSCLC patients harboring EGFR mutations, KRAS mutations, and ALK fusions after initiation of PD-1/PD-L1 blockade treatment, in the 2018 MSKCC database. (C) A box plot evaluating the objective response rate of NSCLC patients, using TIDE prediction scores in the GSE31210 database. (D) A waterfall plot of TIDE prediction scores across 226 NSCLC tumors in the GSE31210 database. Red indicates a tumor that responded to therapy. Blue indicates non-responders. Tumors were divided into 4 categories based on the molecular genotype of NSCLC. In each category, we sorted tumors in descending order according to their TIDE prediction scores. *P < 0.05; **P < 0.01; ***P < 0.001. EGFR mut, EGFR mutation; KRAS mut, KRAS mutation; DCB, durable clinical benefit; Non-DCB, no durable clinical benefit.

To assess the activity of PD-1/PD-L1 inhibitors according to the molecular genotype of NSCLC, we reanalyzed the publicly available trial data (2018 MSKCC), focusing on patients with EGFR, KRAS, and ALK fusion mutations^30^. A total of 207 patients with advanced NSCLC treated with anti-PD-1/PD-L1 monotherapy were included in this analysis. Patient subtypes included: 74 KRAS mutations, 20 EGFR mutations, two ALK fusions, and 111 patients without common mutations. Among patients harboring EGFR mutations or ALK fusions, we observed lower ORRs to PD-1 axis inhibitors when compared with triple wild-type patients. Notably, the ORR in the KRAS-mutant group was the highest of the four molecular subtypes; although, there was no significant difference among patients (**Figure 1A**). Additionally, at the time of survival analysis, we investigated the DCB of patients according to the NSCLC molecular genotype after initiating PD-1/PD-L1 blockade treatment. **Figure 1B** shows that the 6 month PFS rate was lower in the EGFR/ALK-positive groups and higher in the KRAS-positive group compared to the triple negative group.

Based on the above analysis and current literature, anti-PD-1/PD-L1 blockade is probably not applicable for EGFR-mutated and ALK-rearranged NSCLC patients. However, given that there was no publicly available information for analysis on clinical outcomes for East Asian patients treated with PD-1/PD-L1 inhibitors, the TIDE algorithm was used to predict cancer immunotherapy responses in East-Asian patients with this framework and pretreatment RNA-seq data from the GSE31210 dataset^31^. **Figure 1D** shows that the ORRs were 8 of 68 (11.7%) for triple negative patients, 4 of 127 (3.9%) for EGFR-mutant patients, and 3 of 20 (15%) for KRAS-mutant patients. Notably, no patients harboring ALK/EML4 fusions were predicted to respond to anti-PD-1/PD-L1 immunotherapy. The results showed that for anti-PD-1/PD-L1 immunotherapy, EGFR-mutant and ALK-rearranged tumors may result in inferior responses; however, although KRAS-mutant tumors may respond better, the data were not statistically significant (EGFR: *P* = 0.037; ALK: *P* < 0.001; KRAS: *P* = 0.701; **Figure 1C**).

### Meta-analysis of the association between classic driver gene status and PD-L1 expression in NSCLC patients

**Figure 2:**
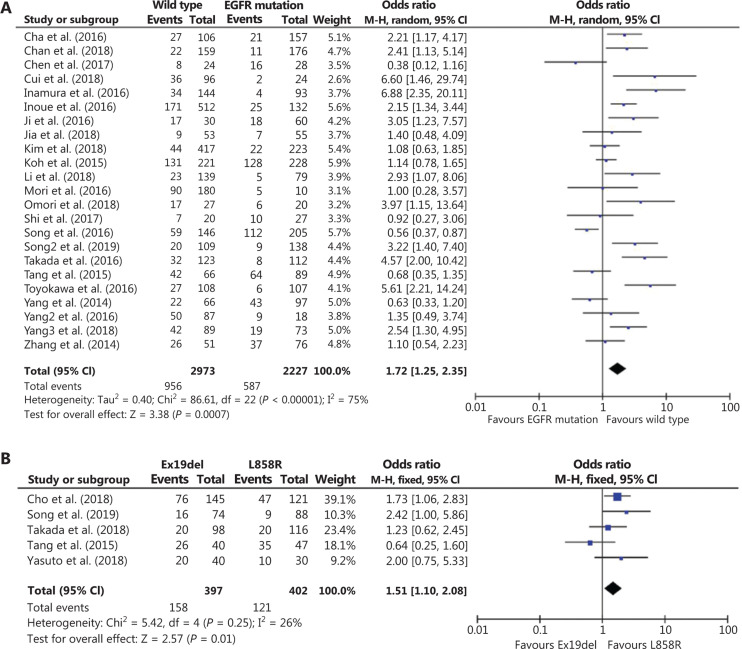
Meta-analysis of the association between PD-L1 expression and EGFR mutation status in non-small cell lung cancer (NSCLC) patients. (A) A forest plot of studies evaluating PD-L1 expression between EGFR wild-type and EGFR mutation patients. Pooled odds ratios of EGFR group analysis were computed using a random-effects model. (B) A forest plot of studies evaluating PD-L1 expression between EGFR Ex19del mutation and EGFR L858R mutation. Pooled odds ratios of EGFR subgroup analyses were computed using a fixed-effects model. CI, confidence interval.

**Figure 3:**
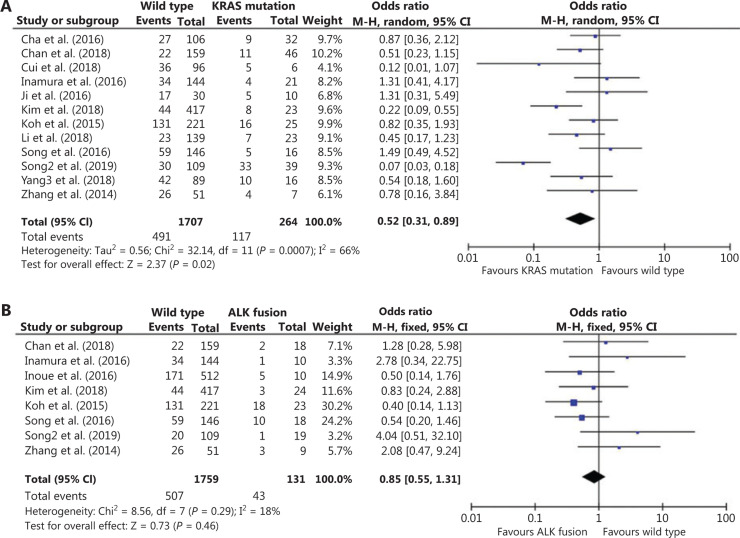
Meta-analysis of the association between PD-L1 expression and KRAS or ALK mutation status in non-small-cell lung cancer (NSCLC) patients. (A) A forest plot of studies evaluating PD-L1 expression between KRAS wild-type and KRAS mutation patients. Pooled odds ratios of KRAS group analysis were computed using a random-effects model. (B) A forest plot of studies evaluating PD-L1 expression between ALK wild-type and ALK fusion patients. Pooled odds ratios of ALK group analyses were computed using a fixed-effects model. CI, confidence interval.

Because no studies have systematically determined possible correlations between the driver oncogene status and PD-L1 expression in East Asian NSCLC patients, we first performed 3 pooled analyses of 23 studies conducted on the East Asian population. Association analysis between PD-L1 expression and EGFR status included 23 studies with 5,200 patients. Compared to EGFR-mutant tumors, PD-L1 expression was mainly associated with EGFR wild-type tumors [odds ratio (OR): 1.72; 95% confidence interval (CI): 1.25–2.35; *P* = 0.0007; **Figure 2A**]. Association analysis between PD-L1 expression and KRAS status included 12 studies with 1971 patients. **Figure 3A** shows that the frequency of positive PD-L1 expression was marginally significantly higher for NSCLC patients with KRAS mutations (OR = 0.52; 95% CI: 0.31–0.89; *P* = 0.02; **Figure 3A**). Eight studies with 1,890 patients were included for association analysis between PD-L1 expression and ALK status. The results showed no significant correlation between PD-L1 expression and ALK rearrangement status (OR = 0.85; 95% CI: 0.55–1.31; *P* = 0.46; **Figure 3B**).

### Correlation between classic driver oncogene mutations and TIME according to the presence of PD-L1 and TIL in NSCLC patients

**Figure 4:**
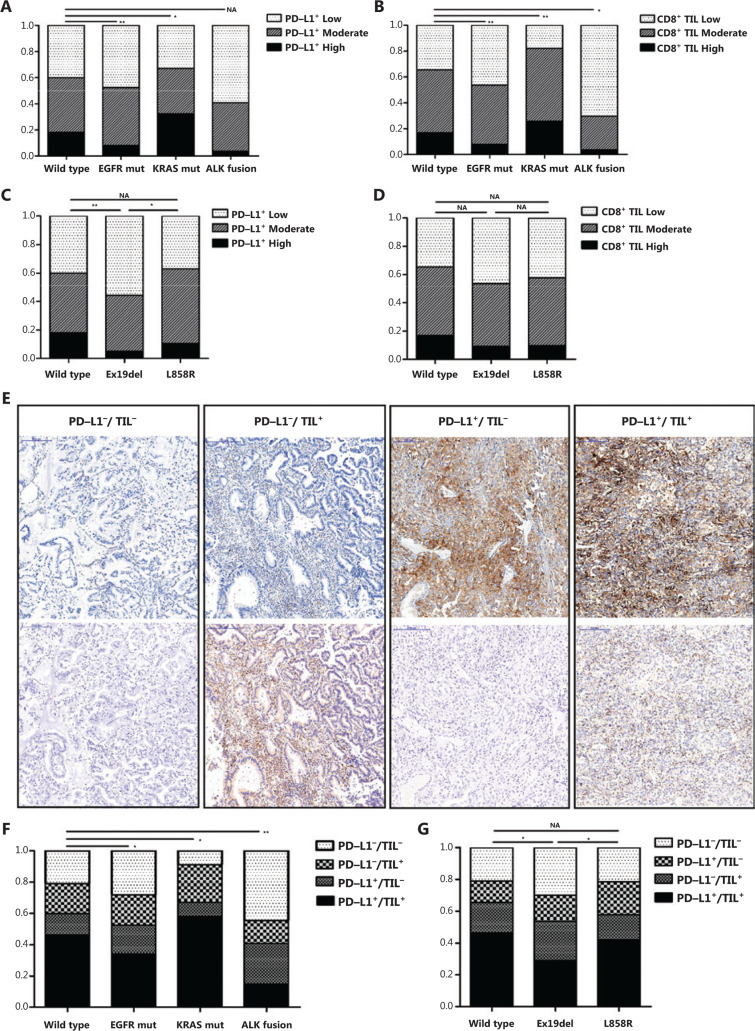
Correlation between the tumor microenvironment based on PD-L1 and CD8^+^ T cell infiltration and classic driver oncogene mutations in non-small-cell lung cancer (NSCLC) patients. (A and B) Immunohistochemical (IHC) analysis of PD-L1 expression (A) and CD8^+^ T cell infiltration (B) according to molecular genotype of NSCLC in a cohort of 629 resected NSCLC samples. (C and D) IHC analyses of PD-L1 expression (C) and CD8^+^ T cell infiltration (D) according to EGFR mutation status. (E) Representative IHC images show classifications of tumor microenvironments based on PD-L1 expression and CD8^+^ T cell infiltration. Scale bar = 200 ?m. (F and G) IHC analysis of the tumor microenvironment based on PD-L1 and CD8^+^ T cell infiltration according to the molecular genotype of NSCLC (F) and EGFR mutation status (G). PD-L1^−^/TIL^−^: PD-L1 TPS < 1% and CD8^+^ TIL density < 1%; PD-L1^+^/TIL^−^: PD-L1 TPS ≥ 1% and CD8^+^ TIL density < 1%; PD-L1^−^/TIL^+^: PD-L1 TPS < 1% and CD8^+^ TIL density ≥ 1%; PD-L1^+^/TIL^+^: PD-L1 TPS ≥ 1% and CD8^+^ TIL density ≥ 1%. *P < 0.05, **P < 0.01. EGFR mut, EGFR mutation; KRAS mut, KRAS mutation.

To further validate these results, IHC detection of PD-L1 was conducted to analyze 629 surgically resected specimens. The clinicopathological characteristics of patients, classic driver oncogene mutation status, and PD-L1 tumor proportion score (TPS) are listed in **Supplementary Table S4**. Compared to the wild-type group, the KRAS-mutant group showed a higher frequency of PD-L1 positivity, demonstrating a positive correlation between KRAS mutation status and PD-L1 expression (*P* = 0.023; **Figure 4A**). However, a lower proportion of PD-L1 strongly positive cells was observed in NSCLC patients harboring EGFR mutations than wild-type patients, revealing a negative correlation between EGFR mutation status and PD-L1 expression (*P* = 0.003; **Figure 4A**). Additionally, although we found NSCLC patients with ALK fusions to have a lower proportion of positive PD-L1 expressions, it was not statistically significant (*P* = 0.077; **Figure 4A**).

Numerous studies have indicated that the presence of TILs is a crucial predictive factor for the efficacy of PD-1 blockade immunotherapy. We therefore investigated the possible correlation between classic driver oncogene alteration status and CD8^+^ T-cell infiltration. A greater abundance of CD8^+^ TILs was found in KRAS-mutant tumors (*P* = 0.006; **Figure 4B**), which was confirmed by IHC analyses of CD8^+^ TILs in 629 resected NSCLC specimens. However, EGFR-mutant or ALK-rearranged tumors showed less T-cell infiltration than triple wild-type tumors (EGFR mutation: *P* = 0.002; ALK fusion: *P* = 0.021; **Figure 4B**).

**Figure 4E** shows that the TIME was classified into four types: PD-L1^−^/TIL^−^ (suggesting immune ignorance); PD-L1^−^/TIL^+^ (suggesting that other suppressors facilitate immune tolerance); PD-L1^+^/TIL^−^ (suggesting intrinsic induction); and PD-L1^+^/TIL^+^ (suggesting adaptive immune resistance). We then determined whether classic driver oncogene mutations influenced the tumor immune microenvironment. The combined analyses of PD-L1 and CD8^+^ TILs showed a significantly higher proportion of dual-positive (PD-L1^+^/TIL^+^) patients in the KRAS-mutant group than in the triple wild-type group (*P* = 0.019; **Figure 4F**), indicating KRAS-mutant lung cancers drove an inflammatory phenotype with adaptive immune resistance. However, the EGFR-mutant or ALK-rearranged groups showed a remarkably higher proportion of PD-L1^−^/TIL^−^ tumors and a lower proportion of PD-L1^+^/TIL^+^ tumors when compared to the triple wild-type patients (EGFR mutation: *P* = 0.044; ALK fusion: *P* = 0.001; **Figure 4F**), suggesting an uninflamed phenotype with immunological ignorance.

### Correlation between EGFR mutation status and the TIME based on the presence of PD-L1 and TILs in NSCLC patients

Although clinical evidence indicates that EGFR-mutant lung cancers rarely benefit from anti-PD-1/PD-L1 immunotherapy^5,9,34^, recent studies, such as ATLANTIC and IMpower150, have reported more positive results for PD-1/PD-L1 inhibitors in EGFR-mutant lung cancers^35,36^. In East Asia, the prevalence of lung cancer with EGFR mutations is more common with a proportion of 30%–40%^21^. Hence, it is vital to determine whether a subgroup of these lung cancer patients could clinically benefit from anti-PD-1/PD-L1 antibodies, and if the TIME subtypes are predictors in such patients. A pooled analysis was performed to assess potential differences in PD-L1 expressions among different EGFR mutations [exon 19 deletion (Ex19del) and codon 858 mutation in exon 21 (L858R)]. The results indicated that NSCLC patients harboring EGFR L858R mutations tended to have a higher frequency of positive PD-L1 expression compared to those harboring EGFR Ex19del mutations (OR = 1.51; 95% CI: 1.10–2.08; *P* = 0.01; **Figure 2B**). To confirm these results, 313 surgically resected EGFR tumors were analyzed using IHC detection for PD-L1. The clinicopathological characteristics of patients, EGFR mutation status, and PD-L1 TPS are listed in **Supplementary Table S5**. The results showed that PD-L1 positivity in the L858R-mutant group was higher compared to that of the Ex19del-mutant group, but similar for the wild-type group (**Figure 4C**). IHC analyses of CD8^+^ TILs in the EGFR-mutant tumors showed no significant differences among these three groups (**Figure 4D**). Furthermore, a remarkably higher proportion of dual-positive samples (PD-L1^+^/TIL^+^) was observed in the L858R-mutant group compared to the Ex19del-mutant group (*P* < 0.05; **Figure 4G**), suggesting an inflammatory phenotype with adaptive immune resistance in EGFR L858R-mutant tumors.

### Correlation between classic driver oncogene mutations and intratumoral immune cell composition in NSCLC patients

**Figure 5:**
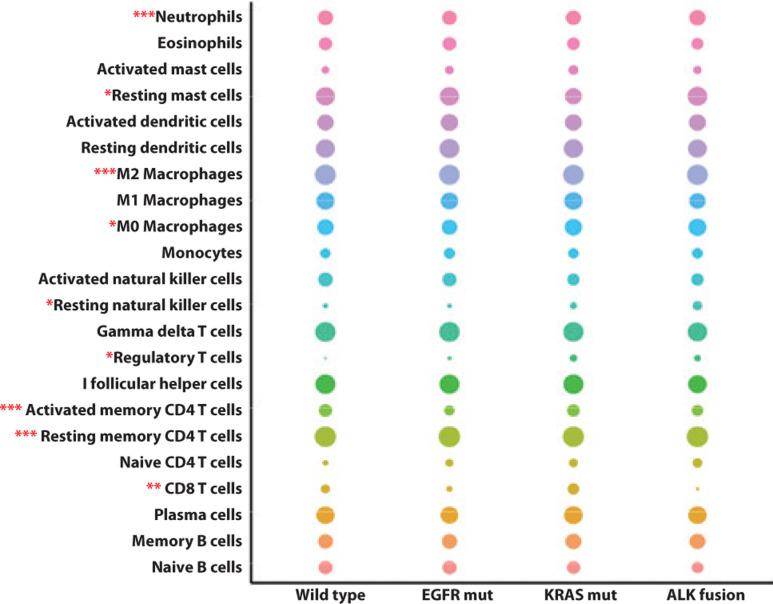
Correlation between infiltrated immune cell composition and classic driver oncogene mutations in non-small cell lung cancer (NSCLC) patients. The size of the bubble represents the numeric value of immune cell fraction. *P < 0.05; **P < 0.01; ***P < 0.001. EGFR mut, EGFR mutation; KRAS mut, KRAS mutation.

**Figure 6:**
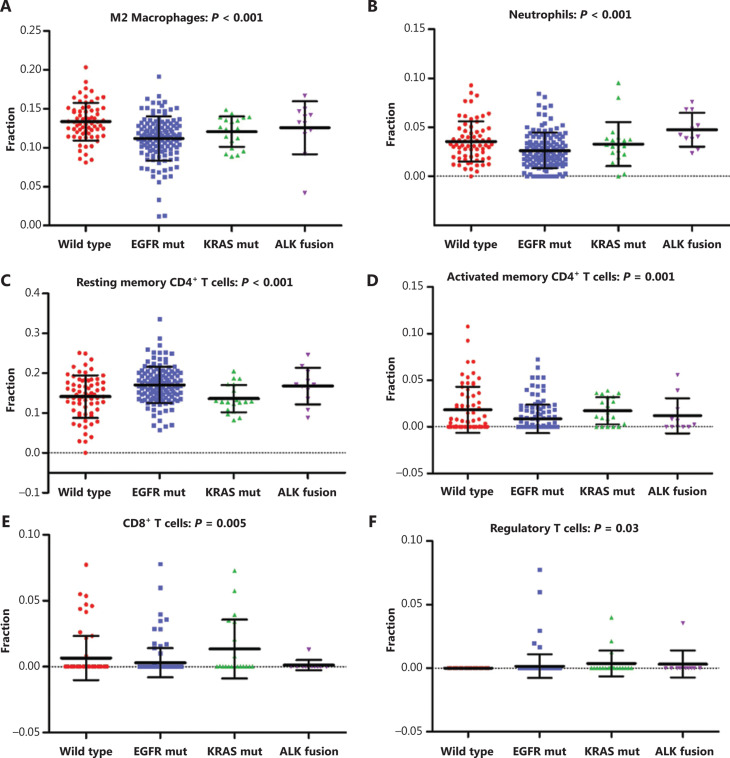
Expression of 6 infiltrated immune cells in 4 molecular subgroups of non-small cell lung cancer (NSCLC) patients. (A–F) Scatter plots of the expression of the specific immune cell types, including M2 macrophages (A), neutrophils (B), resting memory CD4^+^ T cells (C), activated memory CD4^+^ T cells (D), CD8^+^ T cells (E), and regulatory T cells (F), among molecular subgroups of NSCLC patients. EGFR mut, EGFR mutation; KRAS mut, KRAS mutation.

Tumor cells exist in a very complex microenvironment consisting of a diversity of immune cells that interact with tumor cells to ultimately induce tumor cell death or survival. Illuminating the intricacies of tumor immune landscapes may uncover underlying mechanisms of drug resistance to immunotherapy. To identify the distinct immune cell composition in NSCLC molecular subgroups, CIBERSORT, a deconvolution algorithm, was used to calculate the proportions of 22 tumor-infiltrated immune cells in each sample based on gene expression profiles. After filtering using the CIBERSORT P-value, the relative proportions of 22 human leukocyte subsets in different groups are shown in **Figure 5**. Neutrophils, resting mast cells, M2 macrophages, M0 macrophages, resting natural killer cells, regulatory T cells, activated memory CD4^+^ T cells, resting memory CD4^+^ T cells, and CD8^+^ T cells were significantly different among molecular subgroups of NSCLC (*P* < 0.05). **Figure 6** shows further investigation into the specific immune cell types, which revealed that in addition to enriched CD8^+^ T cells, KRAS-mutant NSCLC showed a similar intratumoral immune profile to triple wild-type NSCLC patients. Strikingly, EGFR-mutant and ALK-rearranged tumors were characterized by enriched resting memory CD4^+^ T cells (*P* < 0.001), along with a lack of activated memory CD4^+^ T cells (*P* = 0.001). In the context of tumor genomics, differences in the local repertoire of tumor-infiltrating leukocytes might provide clues to potential mechanisms responsible for the molecular heterogeneity of responses to immunotherapy.

## Discussion

Immune checkpoint inhibitors targeting the PD-1/PD-L1 axis, which allow a patient’s own T cells to kill tumors, are revolutionizing the treatment pattern for numerous cancer, including NSCLC^6^. In this study, we evaluated the correlation between anti-PD-1/PD-L1 immunotherapy efficacy and classic driver oncogene mutations in East Asian NSCLC patients. Our results showed that EGFR-mutant and ALK-rearranged tumors may yield an inferior response than other tumors through an uninflamed phenotype with immune ignorance. Moreover, KRAS-mutant patients appeared to respond better to anti-PD-1/PD-L1 immunotherapy, conferring an inflammatory phenotype with adaptive immune resistance. Notably, EGFR-mutant subtype analysis showed that EGFR L858R-mutant tumors might also have a good outcome with PD-1/PD-L1 blockade through increased PD-L1 expression and a higher proportion of PD-L1^+^/TIL^+^.

Current studies have indicated that the efficacy of PD-1/PD-L1 inhibitors varies among different classic driver oncogene mutations. It was reported that the ORR in EGFR mutation or ALK rearrangement cohorts treated with pembrolizumab was 3.6%, while it was 23.3% in the EGFR/ALK wild-type cohort. Likewise, with respect to OS in the EGFR mutation cohorts, neither pembrolizumab nor nivolumab showed superiority over docetaxel in clinical trials (Checkmate 057 and Keynote-010)^7,9^. Contrary to EGFR/ALK, KRAS mutations were related to better outcomes using PD-1/PD-L1 inhibitors^5,32^. Moreover, our analysis of the 2018 MSKCC trail data also revealed a similar result, showing that KRAS-mutant NSCLC achieved the best efficacy while EGFR-mutant NSCLC showed the lowest response rate in all groups.

Previous studies and publicly available trial data are all based on patients from Europe and the United States. However, it has been reported that lung cancer patients of Asian and Western countries differ not only in histological types but also in genetic mutations. For example, the EGFR mutation rate in patients with lung adenocarcinoma in Europe and the United States is only 10%–17%, but in Asian patients it is 30%–65%. In contrast, the rate of KRAS mutations in the Western Caucasian population is 35%–50%, but it is reported to be less than 5%–10% in Chinese patients^20,37^. Notably, there is currently no systematic study to evaluate the treatment effect of immunotherapy based on the genetic mutation background in Asian populations. In this study, we retrospectively investigated response patterns among EGFR-mutant, KRAS-mutant, ALK-positive, and EGFR wild-type/KRAS wild-type/ALK-negative patients in Asia. We also analyzed subgroups of EGFR-mutant NSCLC patients who may derive clinical benefits from anti-PD-1/PD-L1 agents. The results provided novel evidence of the association between classic driver oncogene mutation status and tumor immunity-associated features in East Asian NSCLC patients.

It is important to note that tumor genotype largely determines its immunophenotype^24^. Previous studies showed that EGFR-mutant NSCLC has low levels of both PD-L1 and CD8^+^ TILs within the tumor microenvironment^38^, and that EGFR mutations associate with uninflamed phenotypes and weak immunogenicity^34^. This could be the reason that EGFR-mutated NSCLC has an inferior clinical response to PD-1-axis immunotherapy. Moreover, a recent study reported that PD-L1 was induced by expression of mutant EGFR in bronchial epithelial cells, and EGFR inhibitors could reduce PD-L1 expression in NSCLC cell lines of activated EGFR^25^. Intriguingly, some studies showed that PD-L1 expression predicts the PFS and OS in NSCLC patients treated with EGFR-TKI^39,40^. However, the benefit of single agent PD-1/PD-L1 inhibitors as second-line treatment or the above treatments is limited in patients with EGFR mutations, because PD-L1 expression is increased but without a better effect^5,34,41^. It has been reported that few CD8^+^ T cells are infiltrating in EGFR-mutant tumors at the same time^34^, which may be the reason for its lack of efficacy. In contrast, with EGFR-mutant tumors, those with KRAS mutations tend to express higher levels of PD-L1^42^ and an enrichment of CD8^+^ T cells^43^. All these results suggest that patients with KRAS mutations may respond favorably to anti-PD-1/PD-L1 immunotherapy. Moreover, recent data showing that checkpoint inhibitors could be more effective in smokers in which somatic gene mutations are frequent, suggest potentially different PD-1/PD-L1 expressions in the presence of some specific molecular events such as KRAS mutations^44,45^. For the TIME based on PD-L1 expression and TILs in ALK-rearranged NSCLC, few studies with larger sample sizes have been reported.

Considering that different genetic backgrounds, geographical distributions, and population lifestyles might cause different PD-L1 expressions, a meta-analysis of PD-L1 expression in Asian NSCLC patients was performed (**Figures 2 and 3**). In addition, we verified our results with IHC analyses (**Figure 4**), demonstrating that PD-L1 expression had a positive correlation with KRAS mutations but a negative correlation with EGFR and ALK mutations. Also, the KRAS-mutant group had a remarkably higher proportion of PD-L1^+^/TIL^+^, while a higher proportion of PD-L1^−^/TIL^−^ and lower proportion of PD-L1^+^/TIL^+^ were observed in EGFR-mutant and ALK-rearranged groups. However, an effort should be made to prospectively validate the association between the TIME and classic driver oncogene mutations in patients who received immune check point inhibitors.

As previously mentioned, recent studies have reported more encouraging results for PD-1/PD-L1 inhibitors in EGFR-mutant lung cancers^35,36^. It was reported that EGFR-mutant tumors commonly have low responses to immune checkpoint blockade, but outcomes differ by allele. Outcomes with PD-1/PD-L1 inhibitors were worse in patients with lung tumors harboring alterations in exon 19 of EGFR, when compared to EGFR wild-type and L858R-mutant lung cancers^46^. Notably, the outcomes of anti-PD-1/PD-L1 immunotherapy contrasted with those on EGFR TKIs, where L858R-mutant tumors had a worse durability of response to EGFR TKIs compared with Ex19del-mutant tumors, highlighting the context specificity of genotypic responses to different therapeutic agents^47,48^. In addition, Hastings et al.^46^ reported that compared with EGFR L858R mutations, lung tumors with EGFR Ex19del alterations harbored a lower tumor mutation burden; yet PD-L1 expression was comparable across EGFR alleles. Our pooled analysis and IHC detection of our clinical cohort showed a higher frequency of positive PD-L1 expression, as well as a higher proportion of PD-L1^+^/TIL^+^ in the EGFR L858R-mutant group. This distinction was likely due to the racial heterogeneity of the study populations. In summary, the present study showed that East Asian NSCLC patients harboring EGFR L858R mutations were associated with an inflammatory tumor microenvironment, which may result in superior patient response to PD-1 inhibitors. These findings provide a basis for further investigating which patients with EGFR mutant disease may be likely to obtain benefits from immunotherapies, especially when combined with chemotherapy or anti-angiogenesis agents. However, the detailed mechanistic explanation between EGFR mutation subtypes and PD-L1 expression needs further research.

Although our understanding of the NSCLC immune landscape has greatly improved, it remains unknown how the local repertoire of tumor infiltrating leukocytes differs according to tumor genomics, and the impact it has on prognosis and response to immunotherapy. This justified our examination of immune cell elements from bulk tissue GEPs, and our characterization of the individual immune cell composition in molecular subgroups of NSCLC. As shown in **Figure 5**, there was a significant difference in the local repertoire of tumor-infiltrating leukocytes among molecular subgroups of NSCLC. These data could offer clues to potential mechanisms responsible for immunotherapy responses based on molecular heterogeneity. Additionally, we found that EGFR-mutant and ALK-rearranged NSCLC showed a different intratumoral immune profile than other tumors, characterized by a lack of CD8^+^ and activated memory CD4^+^ T cells, as well as an enrichment of resting memory CD4^+^ T cells. This explained why EGFR-mutant and ALK-rearranged NSCLC may not benefit from anti-PD-1/PD-L1 immunotherapy. Efforts should be made to confirm these findings in independent patient cohorts, and further studies should seek methods for improving the immunosuppressive microenvironment of EGFR-mutant and ALK-rearranged tumors.

## Conclusions

For the first time, we have demonstrated that molecular heterogeneity in the response to immunotherapy in East Asian NSCLC patients was correlated with the tumor immune microenvironment based on PD-L1 expression, TIL presence, and the local repertoire of tumor infiltrating leukocytes. Identifying molecular subtypes that offer predictive value is therefore critical for the appropriate protocol of anti-PD-1/PD-L1 immunotherapy. Further study is required to confirm these relationships in independent patient cohorts and to determine the impact of the immune landscape on immunotherapeutic responses.
