# Supplementary material for: Molecular heterogeneity of anti-PD-1/PD-L1 immunotherapy efficacy is correlated with tumor immune microenvironment in East Asian patients with non-small cell lung cancer
Source: Cancer Biol Med. 2020 Aug 15;17(3):768–81. doi: 10.20892/j.issn.2095-3941.2020.0121 (PMC7476088; doi:10.20892/j.issn.2095-3941.2020.0121)
Supplement: Supplementary file 1 [file cbm-17-768-s001.pdf]

Supplementary materials

Table S1 Demographic characteristics and their correlations with clinical outcomes in a cohort of 207 advanced NSCLC patients treated with anti-PD-1/PD-L1 monotherapy

Characteristics	Whole population	Wild type	EGFR mutation	KRAS mutation	ALK fusion	*P*
Case no.	207	111	20	74	2	
Gender, n (%)						0.294
Male	101 (48.8)	61 (55.0)	8 (40.0)	31 (41.9)	1 (50.0)	
Female	106 (51.2)	50 (45.0)	12 (60.0)	43 (58.1)	1 (50.0)	
Age, n (%)						0.368
<60	63 (30.4)	34 (30.6)	9 (45.0)	19 (25.6)	1 (50.0)	
≥ 60	144 (69.6)	77 (69.4)	11 (55.0)	55 (74.3)	1 (50.0)	
Smoking status, n (%)						<0.001
Never	38 (18.4)	24 (21.6)	9 (45.0)	4 (5.4)	1 (50.0)	
Ever	169 (81.6)	87 (78.4)	11 (55.0)	70 (94.6)	1 (50.0)	
Therapy line, n (%)						0.545
1st line	33 (15.9)	16 (14.4)	2 (10.0)	15 (20.3)	0 (0)	
≥ 2nd line	174 (84.1)	95 (85.6)	18 (90.0)	59 (79.7)	2 (100.0)	
Objective response, n (%)						0.453
PR	39 (18.9)	20 (18.0)	2 (10.0)	17 (23.0)	0 (0)	
SD	67 (39.3)	40 (36.0)	4 (20.0)	22 (29.7)	1 (50.0)	
PD	101 (48.8)	51 (46.0)	14 (70.0)	35 (47.3)	1 (50.0)	
Durable clinical benefit, n (%)						0.059
YES	53 (25.6)	29 (26.1)	2 (10.0)	22 (29.7)	0 (0)	
NO	143 (69.1)	77 (69.4)	16 (80.0)	49 (66.2)	1 (50.0)	
Unknown	11 (5.3)	5 (4.5)	2 (10.0)	3 (4.1)	1 (50.0)	

Table S2 Basic characteristics of studies focused on the correlation between PD-L1 expression and classic driver gene status in NSCLC patients

Study ID	Region	Number of patients				Histology	Stage	Method (Ab)	Definition of PD-L1⁺	Number of PD-L1⁺ patients			
		Wild type	EGFR mut	KRAS mut	ALK fusion					Wild type	EGFR mut	KRAS mut	ALK fusion
Cha et al. (2016)	Korea	106	157	32	–	Mix	I-IV	IHC (clone SP142)	≥ 5%	27	21	9	–
Chan et al. (2018)	China	159	176	46	18	Mix	I-IV	IHC (clone 22C3)	≥ 50%	22	11	11	2
Chen et al. (2017)	China	24	28	–	–	ADC	I-III	IHC (17952-1-AP)	≥ 5%	8	16	–	–
Cui et al. (2018)	China	96	24	6	–	Mix	I-IIIA	IHC (clone E1L3N)	≥ 5%	36	2	5	–
Inamura et al. (2016)	Japan	144	93	21	10	ADC	I-IV	IHC (clone E1L3N)	≥ 5%	34	4	4	1
Inoue et al. (2016)	Japan	512	132	–	10	Mix	I-III	IHC (clone E1L3N)	≥ 5%	171	25	–	5
Ji et al. (2016)	China	30	60	10	–	ADC	I-III	IHC (ab174838)	≥ 5%	17	18	5	–
Jia et al. (2018)	China	53	55	–	–	Mix	NR	IHC (clone E1L3N)	≥ 10%	9	7	–	–
Kim et al. (2018)	Korea	417	223	23	24	Mix	I-IV	IHC (clone 22C3)	≥ 1%	44	22	8	3
Koh et al. (2015)	Korea	221	228	25	23	ADC	I-III	IHC (clone E1L3N)	≥ 5%	131	128	16	18
Li et al. (2018)	China	139	79	23	–	Mix	I-III	IHC (clone SP263)	≥ 50%	23	5	7	–
Mori et al. (2016)	Japan	180	10	–	–	ADC	I-III	IHC (clone EPR1611)	score 50	90	5	–	–
Omori et al. (2018)	Japan	27	20	–	–	Mix	I-IV	IHC (clone E1L3N)	≥ 1%	17	6	–	–
Shi et al. (2017)	China	20	27	–	–	ASC	I-IV	IHC (clone SP263)	≥ 25%	7	10	–	–
Song et al. (2016)	China	146	205	16	18	ADC	I-III	IHC (clone 5H1)	≥ 5%	59	112	5	10
Song et al. (2019)	China	109	138	39	19	Mix	I-IV	IHC (clone 22C3 or SP263)	≥ 50%	20	9	33	1
Takada et al. (2016)	Japan	123	112	–	–	ADC	I-III	IHC (clone SP142)	≥ 5%	32	8	–	–
Tang et al. (2015)	China	66	89	–	–	Mix	IIIB-IV	IHC (clone E1L3N)	≥ 5%	42	64	–	–
Toyokawa et al. (2016)	Japan	108	107	–	–	Mix	I-III	IHC (clone SP142)	≥ 5%	27	6	–	–
Yang et al. (2014)	China	66	97	–	–	ADC	I	IHC (17952-1-AP)	≥ 5%	22	43	–	–
Yang et al. (2016)	China	87	18	–	–	SCC	I	IHC (17952-1-AP)	≥ 5%	50	9	–	–
Yang et al. (2018)	China	89	73	16	–	Mix	I-IV	IHC (clone E1L3N)	≥ 5%	42	19	10	–
Zhang et al. (2014)	China	51	76	7	9	ADC	I-III	IHC (SAB2900365)	≥ 40% and staining ≥ 2	26	37	4	3

Table S3 Main characteristics of included studies in pool analysis performed to investigate PD-L1 expression according to mutant subtype in EGFR-mutant patients

Study ID	Region	Number of patients		Histology	Stage	Method (Ab)	Definition of PD-L1$^+$	Number of PD-L1$^+$ patients	
		Ex19del	L858R					Ex19del	L858R
Cho et al. (2018)	Korea	145	121	Mix	I-IV	IHC (clone 22C3)	≥ 1%	76	47
Song et al. (2019)	China	74	88	ADC	I-IV	IHC (clone SP263)	≥ 25%	16	9
Takada et al. (2018)	Japan	98	116	ADC	I-V	IHC (clone SP142)	≥ 5%	20	20
Tang et al. (2015)	China	40	47	Mix	IIIB-IV	IHC (clone E1L3N)	≥ 5%	26	35
Yasuto et al. (2018)	Japan	40	30	ADC	III-IV	IHC (clone 22C3)	≥ 1%	20	10

Table S4 Demographic characteristics and their correlations with PD-L1 expression and CD8$^+$ TILs among molecular subgroups of NSCLC

Characteristics	Whole population	Wild type	EGFR mutation	KRAS mutation	ALK/EML4 fusion	*P*
Case no.	629	177	313	112	27	
Gender, n (%)						<0.001
Male	313 (49.8)	111 (62.7)	113 (36.1)	78 (69.6)	11 (40.7)	
Female	316 (50.2)	66 (37.3)	200 (63.9)	34 (30.4)	16 (59.3)	
Age, n (%)						0.105
<60	337 (53.6)	87 (49.2)	171 (54.6)	59 (52.7)	20 (74.1)	
≥ 60	292 (46.4)	90 (50.8)	142 (45.7)	53 (47.3)	7 (25.9)	
Smoking status, n (%)						<0.001
Never	367 (58.3)	77 (43.5)	237 (36.1)	32 (28.6)	21 (77.8)	
Ever	262 (41.7)	100 (56.5)	76 (63.9)	80 (71.4)	6 (22.2)	
Histology, n (%)						<0.001
Adeno	535 (85.1)	120 (67.8)	294 (93.9)	95 (84.8)	26 (96.3)	
Others	94 (14.9)	57 (32.2)	19 (6.1)	17 (15.2)	1 (3.7)	
PD-L1 TPS, n (%)						<0.001
<1%	274 (43.6)	71 (40.1)	149 (47.6)	37 (33.0)	16 (59.3)	
1–49%	261 (41.5)	74 (41.8)	139 (44.4)	39 (34.8)	10 (37.0)	
≥ 50%	94 (14.9)	32 (18.1)	25 (8.0)	36 (32.2)	1 (3.7)	
CD8$^+$ TIL, n (%)						<0.001
<1%	242 (38.5)	61 (34.5)	145 (46.3)	20 (17.8)	19 (70.3)	
1–9%	303 (48.2)	86 (48.6)	144 (46.0)	63 (56.3)	7 (26.0)	
≥ 10%	84 (13.3)	30 (16.9)	24 (7.7)	29 (25.9)	1 (3.7)	
TIME, n (%)						<0.001
PD-L1$^-$/TIL$^-$	147 (23.3)	37 (20.9)	88 (28.1)	10 (8.9)	12 (44.4)	
PD-L1$^-$/TIL$^+$	109 (17.3)	34 (19.2)	61 (19.5)	10 (8.9)	4 (14.8)	
PD-L1$^+$/TIL$^-$	115 (18.3)	24 (13.6)	57 (18.2)	27 (24.1)	7 (26.0)	
PD-L1$^+$/TIL$^+$	258 (41.1)	82 (46.3)	107 (34.2)	65 (58.1)	4 (14.8)	

Table S5 Demographic characteristics and their correlations with PD-L1 expression and $CD8^+$ TILs in a cohort of EGFR-mutant NSCLC patients

Characteristics	EGFR mutation	Ex19del	L858R	Others	*P*
Case no.	313	97	135	81	
Gender, n (%)					0.425
Male	113 (36.1)	38 (39.2)	41 (30.4)	34 (42.0)	
Female	200 (63.9)	59 (60.8)	94 (69.6)	47 (58.0)	
Age, n (%)					0.731
<60	171 (54.6)	59 (60.8)	65 (48.1)	47 (58.0)	
≥ 60	142 (45.7)	38 (39.2)	70 (51.9)	34 (42.0)	
Smoking status, n (%)					0.060
Never	237 (36.1)	71 (73.2)	108 (80.0)	58 (71.6)	
Ever	76 (63.9)	26 (26.8)	27 (20.0)	23 (28.4)	
Histology, n (%)					0.080
Adeno	294 (93.9)	94 (96.9)	130 (96.3)	70 (86.4)	
Others	19 (6.1)	3 (3.1)	5 (3.7)	11 (13.6)	
PD-L1 TPS, n (%)					0.025
<1%	149 (47.6)	54 (55.7)	50 (37.0)	45 (55.5)	
1–49%	139 (44.4)	38 (39.2)	71 (52.6)	30 (37.1)	
≥ 50%	25 (8.0)	5 (5.1)	14 (10.4)	6 (7.4)	
$CD8^+$ TIL, n (%)					0.250
<1%	145 (46.3)	45 (46.4)	57 (42.2)	43 (53.1)	
1–9%	144 (46.0)	43 (44.3)	65 (48.2)	36 (44.4)	
≥ 10%	24 (7.7)	9 (9.3)	13 (9.6)	2 (2.5)	
TIME, n (%)					
$PD-L1^-/TIL^-$	88 (28.1)	29 (29.9)	29 (21.5)	30 (37.0)	0.044
$PD-L1^-/TIL^+$	61 (19.5)	25 (24.7)	21 (15.6)	15 (18.5)	
$PD-L1^+/TIL^-$	57 (18.2)	16 (16.5)	28 (20.7)	13 (16.1)	
$PD-L1^+/TIL^+$	107 (34.2)	27 (28.9)	57 (42.2)	23 (28.4)	
